# Supplementary material for: Large‐scale image‐based profiling of single‐cell phenotypes in arrayed CRISPR‐Cas9 gene perturbation screens
Source: Mol Syst Biol. 2018 Jan 23;14(1):e8064. doi: 10.15252/msb.20178064 (PMC5787707; doi:10.15252/msb.20178064)
Supplement: Supplementary file 1 — Expanded View Figures PDF [file MSB-14-e8064-s001.pdf]

Expanded View Figures

Figure EV1. Functional genetic perturbation of human cells by transient transfection of targeting plasmids.

A Immunofluorescence staining of LAMP1 in HeLa cells transfected with a control plasmid, or a *LAMP1* targeting plasmid. Scale bar, 50 µm. Violin plots of normalized mean LAMP1 staining intensity in tdTomato expressing (T(+)) cells 4 days post-transfection.

B Immunofluorescence staining of YAP1 in HeLa cells transfected with a control plasmid, or a *YAP1* targeting plasmid. Scale bar, 50 µm. Violin plots of normalized mean YAP1 staining intensity in tdTomato expressing (T(+)) cells 4 days post-transfection.

C Immunofluorescence staining of LAMP1 in U2OS cells transfected with a control plasmid, or a *LAMP1* targeting plasmid. Scale bar, 50 µm. Violin plots of normalized mean TFRC staining intensity in tdTomato expressing (T(+)) cells 4 days post-transfection.

D Rational selection of highly functional gRNA sequences, see main text and material and methods for details.

© 2018 The Authors

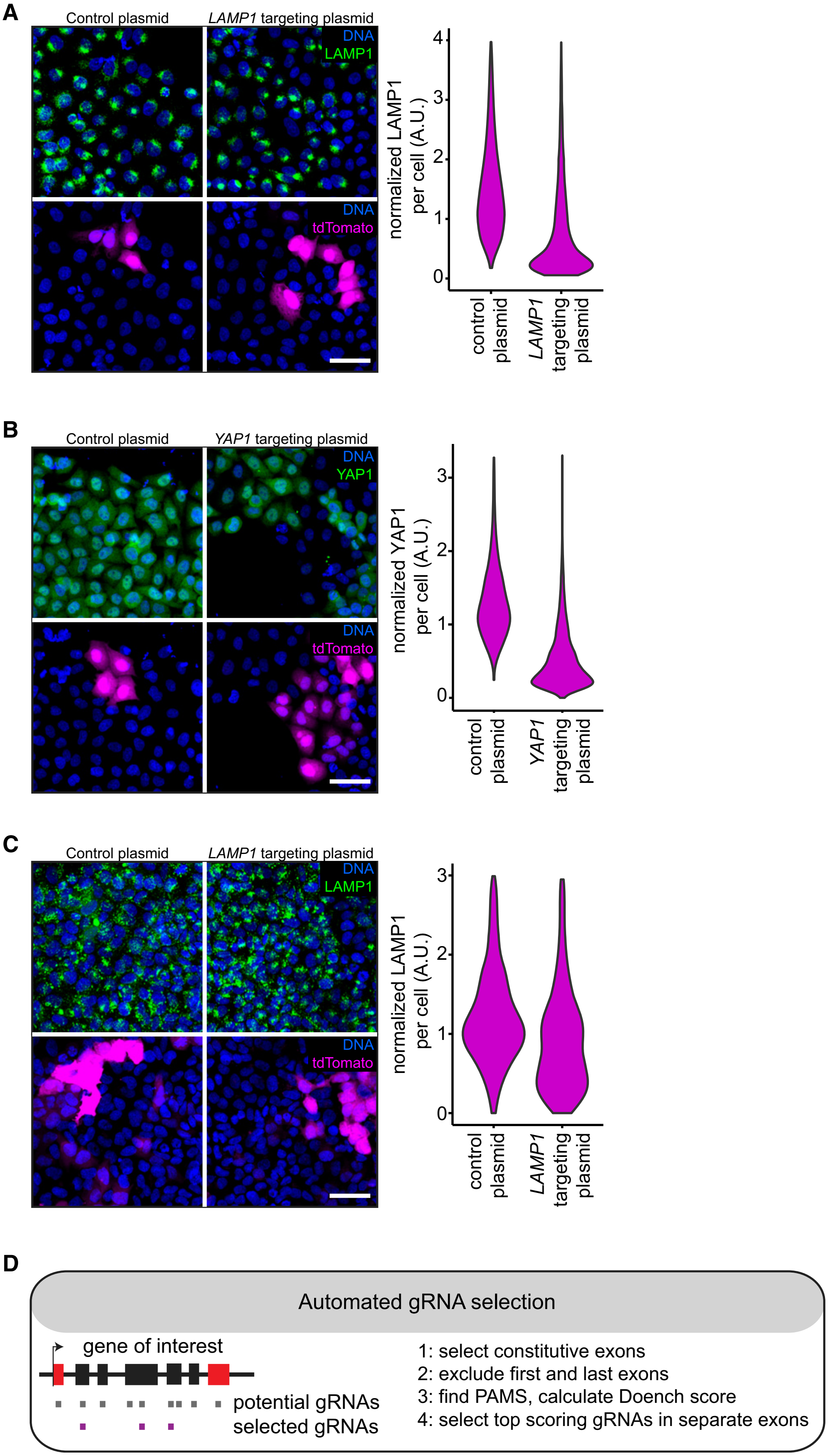

Figure EV1.

© 2018 The Authors

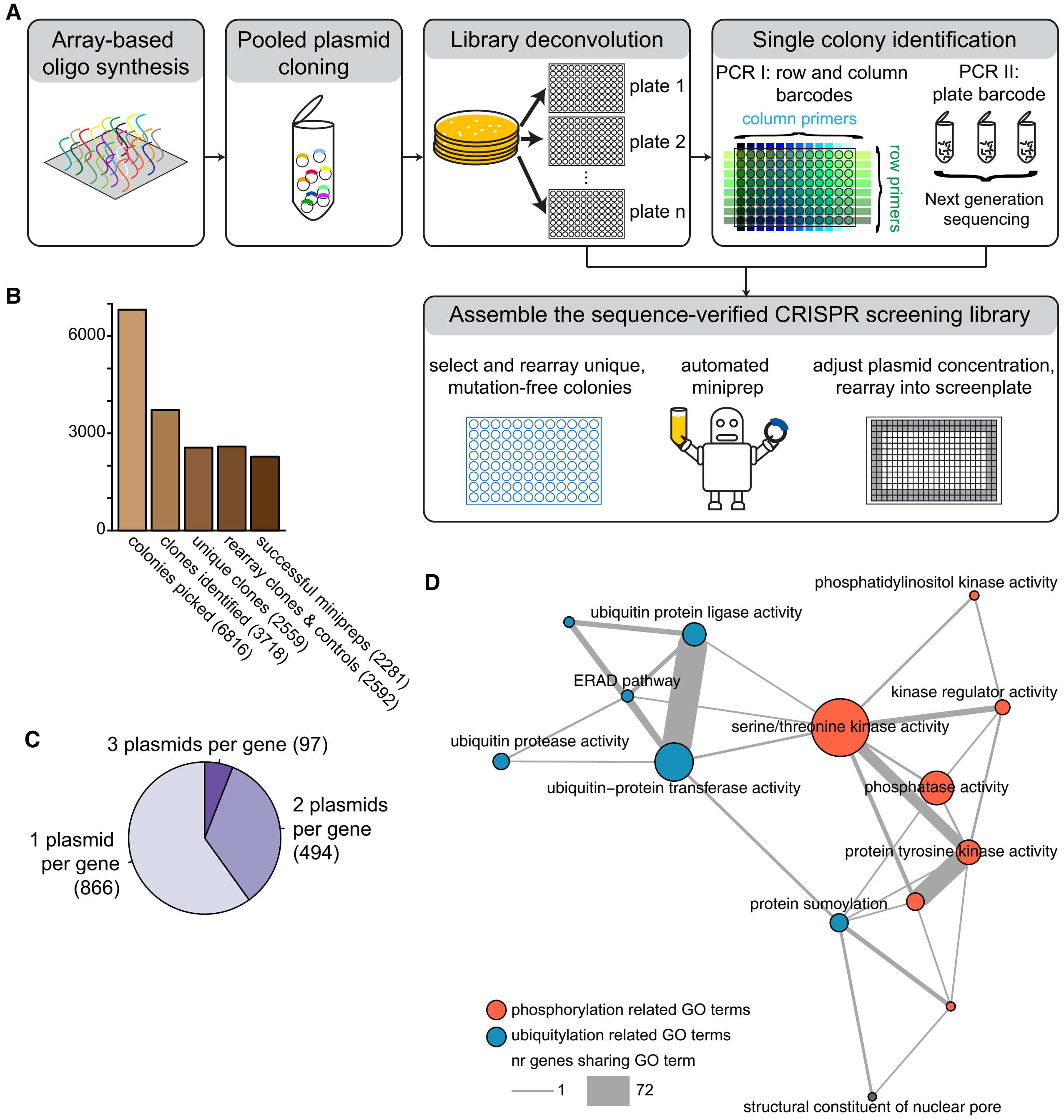

Figure EV2. A large-scale arrayed CRISPR-Cas9 screening library.

A Schematic representation of the workflow for the construction of an arrayed CRISPR-Cas9 screening library. A pool of oligos is synthesized and cloned into the vector backbone in a single reaction. Single colonies are picked into multi-well plates. The gRNA sequence of every colony is PCR amplified with primers that introduce barcodes to identify the row, column and plate of the well where the colony is located. The sequence of the PCR products is analysed in a deep sequencing reaction. Unique, mutation-free colonies are selected, re-arrayed and miniprepped to generate an arrayed CRISPR-Cas9 screening library.

B Representation of the number of picked colonies, the number of mutation-free identified gRNAs, the number of unique mutation-free gRNAs, the number of re-arrayed colonies and the number of constructs in the arrayed CRISPR-Cas9 screening library.

C Pie chart representing the number of genes targeted by 1, 2 or 3 targeting plasmids.

D Network representation of the arrayed CRISPR-Cas9 screening library. Nodes represent selected Gene Ontology annotations of targeted genes, node size represents the number of genes with the functional annotation, edges indicate genes sharing functional annotations. Edge thickness scales with number of genes that share functional annotations. Nodes are colour coded for phosphorylation-related functional annotations (red) or ubiquitylation-related functional annotations (blue).

© 2018 The Authors

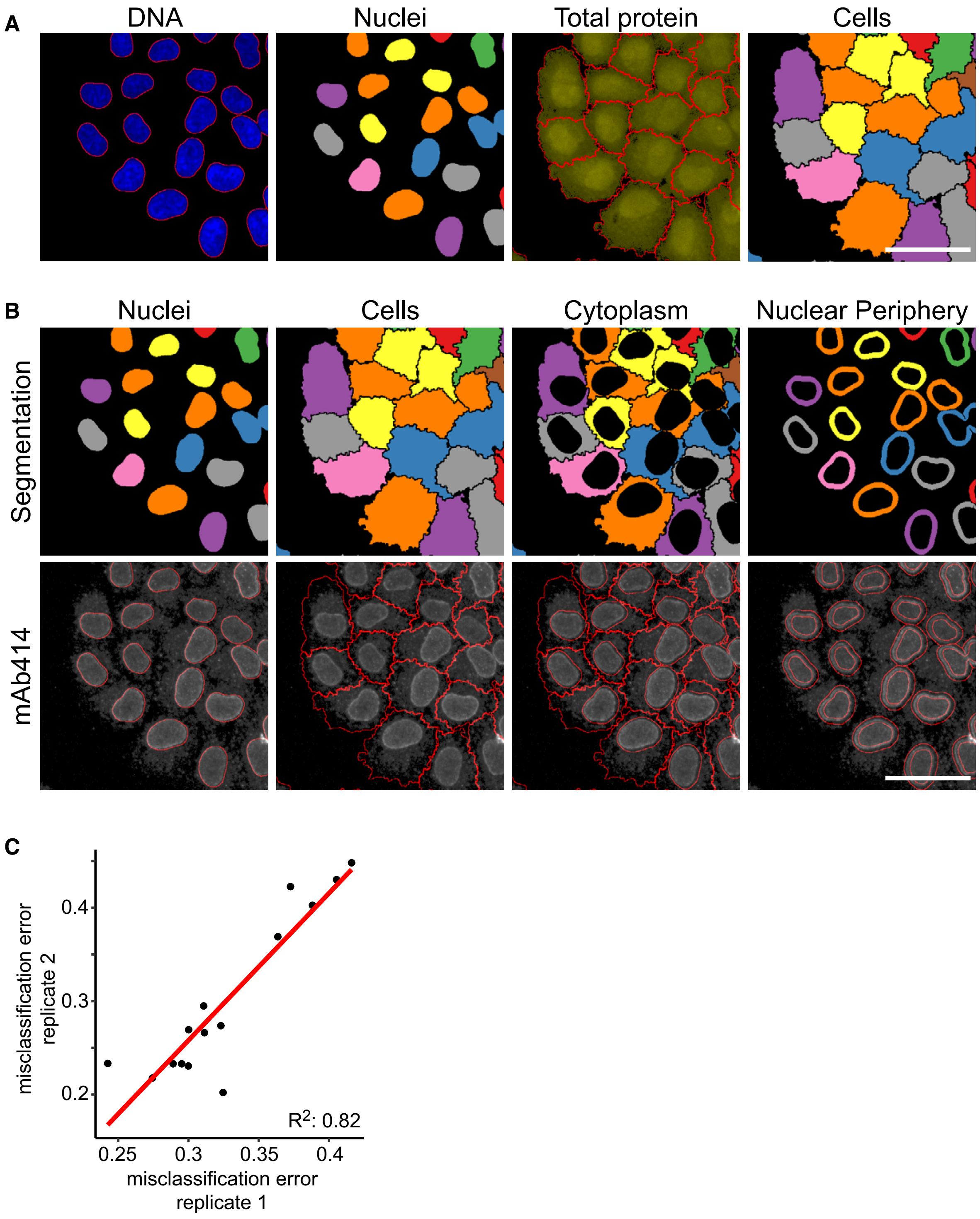

Figure EV3. CRISPR-Cas9 gene perturbation profiling in HeLa cells.

A Nucleus and cell segmentation based on image processing and computer vision of cells stained for DNA and total protein. Scale bar, 50 μm.

B Nucleus, cell, cytoplasm and nuclear periphery segmentation and mAb414 staining for the large-scale CRISPR-Cas9 gene perturbation profiling experiment of the mAb414 staining pattern. Scale bar, 50 μm.

C Scatterplot of misclassification errors of classifiers trained on cells transfected with plasmids targeting *HSPA5*, NPC components and non-targeting controls from two independent experiments.

 © 2018 The Authors

Figure EV4. Large-scale image-based CRISPR-Cas9 gene perturbation profiling.

A Network representation of selected GO terms associated with perturbations identified in the profiling of cell morphology and total protein staining features. Edges between nodes are formed if GO terms share genes. Node size represents enrichment of GO terms relative to the screening library, and the *P*-value is calculated using a hypergeometric test.

B Network representation of GO terms associated with perturbations identified in the profiling of the mAb414 staining pattern. Node size represents enrichment of GO terms relative to the screening library, and *P*-values are calculated using a hypergeometric test.

C Hierarchical clustering of the mean feature values of phenotypically perturbed cells from populations of cells transfected with plasmids targeting proteasome subunits and mean feature profiles of cells transfected with non-targeting control plasmids. The mean feature profiles were calculated based on all features used in the cell morphology profiling.

© 2018 The Authors

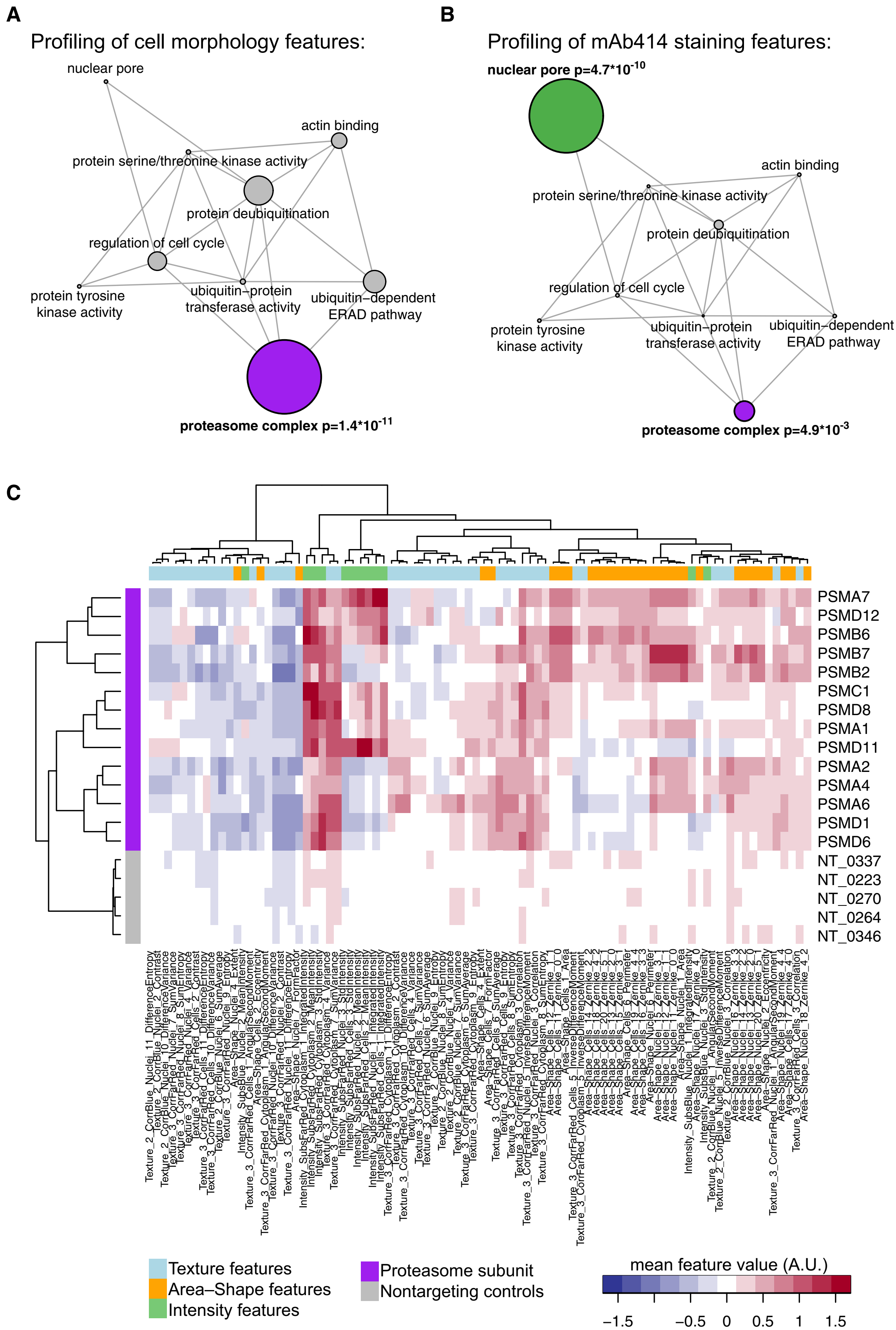

Figure EV4.

 © 2018 The Authors

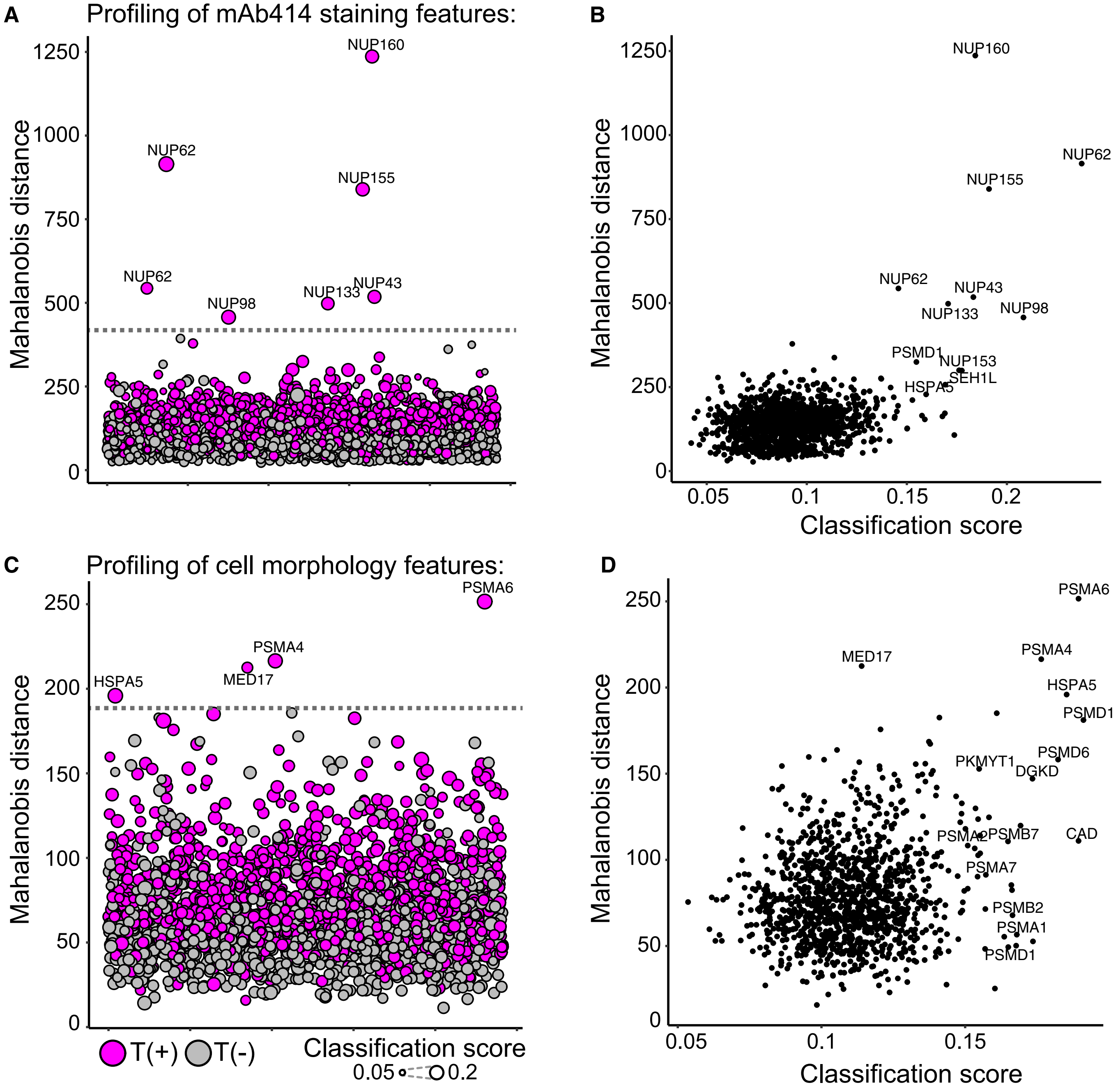

Figure EV5. Phenotypic profiling by between-well comparison of feature profiles.

A, C The mean mAb414 feature profiles (A) or cell morphology features (C) were calculated for T(+) and T(−) cells per well. For each profile, the Mahalanobis distance from the distribution of all profiles was calculated. Nodes represent feature profiles, colour-coded magenta and grey for profiles obtained from T(+) and T(−) cells, respectively. The dotted line indicates the threshold used to select perturbations have a large distance to non-targeting controls (third quartile + 3 × interquartile range of the distance of non-targeting controls). Nodes are scaled according to the classification score which is based on the within-well comparison of T(+) and T(−) cells.

B, D The Mahalanobis distance of T(+) profiles from the total distribution of mean feature profiles was plotted against the classification score (as obtained from within-well comparison of T(+) and T(−) cells) for the profiling of the mAb414 features (B) and cell morphology features (D).

© 2018 The Authors

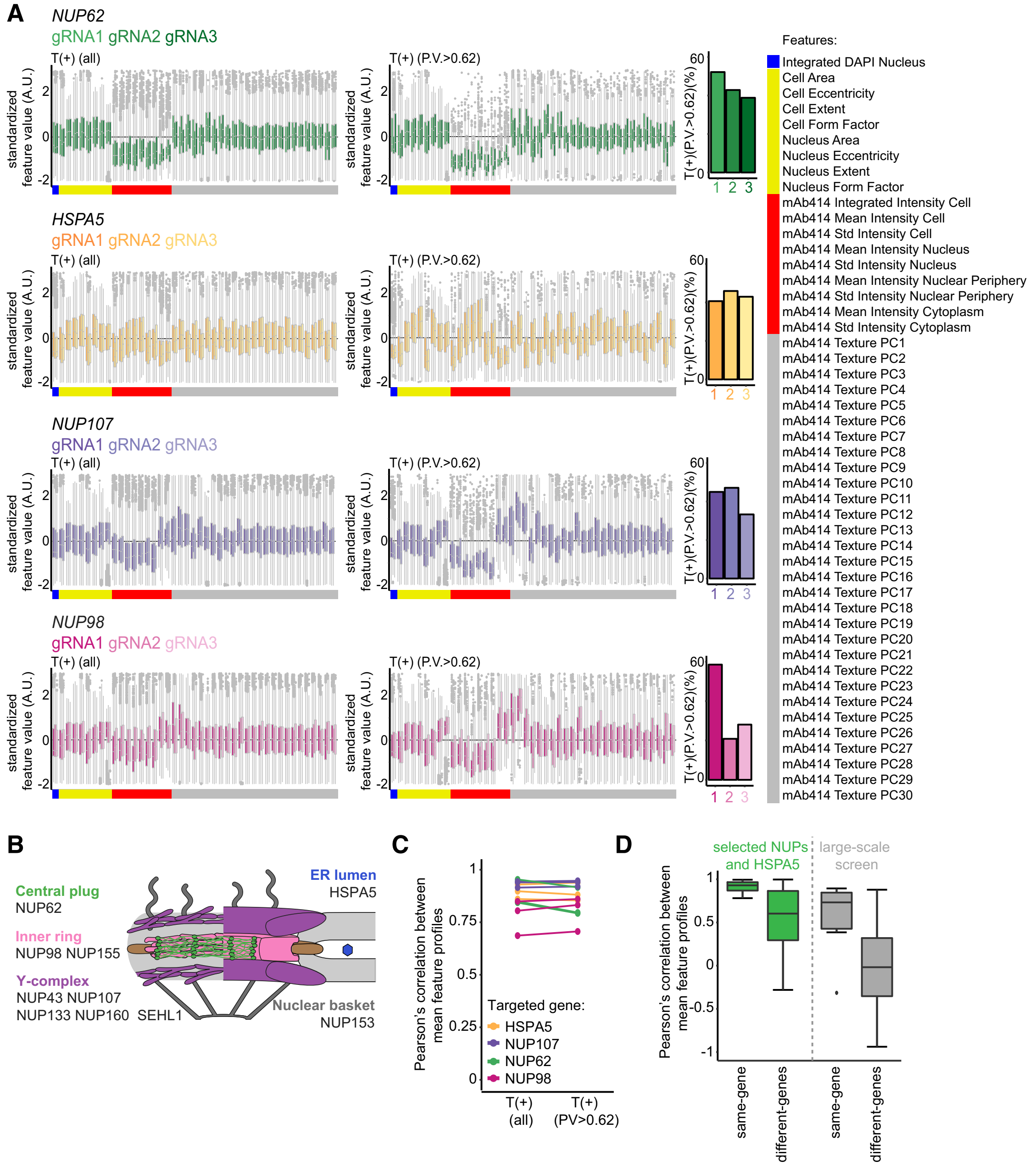

Figure EV6. Mean feature profiles of targeted cells are highly consistent between gRNA sequences.

A Boxplots of the standardized single-cell feature values of all transfected cells and phenotypically perturbed cells transfected with plasmids targeting *NUP62*, *HSPA5*, *NUP107* or *NUP98*, bar graph representation of the percentage of T(+) cells with a PV > 0.62. Boxes indicate the 1st and 3rd quartile of the data distribution. The whiskers indicate the maximum and minimum datapoints within the 1st quartile minus 1.5 times the interquartile range (IQR) of the data and the third quartile plus 1.5 times the IQR.

B Schematic representation of the NPC, adapted from Weberruss and Antonin (Weberruss & Antonin, 2016).

C Cells were transfected with three independent plasmids targeting each of the genes *NUP62*, *HSPA5*, *NUP107* or *NUP98*. Mean feature profiles were obtained from all transfected cells, or the subset of T(+) cells with a high PV. The Pearson correlation coefficient between pairs of profiles obtained from populations targeted for the same gene with different plasmids was calculated. The correlations between profiles obtained from all transfected cells, or the subset of T(+) cells with a high PV are compared.

D Boxplots of Pearson's correlation coefficients calculated between mean feature profiles of phenotypically perturbed cells transfected with plasmids targeting the same gene, or different genes. Phenotypic profiles were obtained from cells transfected with plasmids targeting selected subunits of the NPC and HSPA5 (green) or the top-scoring genes that were identified in the large-scale profiling of the mAb414 staining features for which multiple targeting plasmids were present in the library (grey). Boxes indicate the 1st and 3rd quartile of the data distribution. The whiskers indicate the maximum and minimum datapoints within the 1st quartile minus 1.5 times the interquartile range (IQR) of the data and the third quartile plus 1.5 times the IQR.

 © 2018 The Authors
